# Peer-facilitated treatment access for hepatitis C: the Live Hep C Free project

**DOI:** 10.1186/s12954-022-00619-3

**Published:** 2022-04-21

**Authors:** Julia A. Silano, Carla Treloar, Kyle Leadbeatter, Sandy Davidson, Justine Doidge

**Affiliations:** 1 McMaster University, Hamilton, Canada; 2 University of New South Wales, Sydney, Australia; 3 Hepatitis NSW, Sydney, Australia

**Keywords:** Hepatitis C, Peer workers, Socially marginalised groups, Community-based HCV care

## Abstract

**Background:**

This commentary explores the lessons learned during implementation of a peer-facilitated hepatitis C virus (HCV) testing and treatment access project called the Live Hep C Free (LHCF) project in contributing to micro-elimination efforts.

**Case presentation:**

The LHCF project aims to facilitate access to on-the-spot HCV testing, treatment, and care in priority settings through partnership between a peer worker (PW) and a clinical nurse. Since the start of the project in January 2018, 4515 people were engaged about HCV and encouraged to access on-site HCV health care, and over 1000 people were screened for HCV and liver health, while almost 250 people accessed HCV treatment through the project. This commentary is intended to prompt discussion about incorporating peer-centred HCV health programs into priority sites. HCV care-delivery models such as the LHCF project can continue to contribute to micro-elimination of HCV in key settings to increase treatment uptake amongst high prevalence and/or marginalised populations and support progress toward national elimination targets.

**Conclusions:**

The LHCF project has been able to highlight the benefits of incorporating trustworthy, efficient, and convenient peer-centred health services to engage and support vulnerable populations through HCV testing and treatment, particularly individuals who have historically been disconnected from the health care system. Additional attention is needed to ensure ongoing funding support to sustain the project and deliver at scale and in expanding evaluation data to examine the operation and outcomes of the project in more detail.

## Background

Most infectious diseases disproportionately impact marginalised populations; a fact that is especially true for HCV in Australia [1]. Despite accessible direct acting antiviral (DAA) medications in Australia, low rates of HCV treatment uptake among marginalised populations persist [2, 3]. Barriers to HCV diagnosis and treatment uptake among marginalised populations in Australia, such as people experiencing homelessness, from culturally and linguistically diverse backgrounds, accessing drug treatment programs, as well as people who inject drugs or have a history of injecting drug use and Aboriginal and Torres Strait Islander people [4], are multifactorial. Personal barriers to accessing HCV care have been cited to include being asymptomatic, finding the right time for DAA treatment and poor vein health [3]. System-level barriers include managing multiple health and social priorities such as finding transportation and/or childcare when attending treatment appointments [3]. Finding supportive and non-judgmental care is often difficult for marginalised populations and system-level barriers often perpetuate stigmatising and discriminatory attitudes and behaviours on behalf of clinical staff and health practitioners [3]. Understanding barriers to accessing testing and treatment are critical to ensuring that HCV community health care efforts are sensitive, effective, and equitable.

Considering the burden of disease and the barriers to accessing HCV care, concerted efforts to provide comprehensive and innovative models of health care delivery are required [4]. Community-based programs that provide comprehensive screening, diagnosis, and linkage services for people at high risk for HCV may improve engagement across the continuum of care [5]. Recently, strategic programming efforts to maximise HCV elimination efforts have shifted to incorporate peer-focused outreach initiatives. Peer support models are an effective approach in terms of assessment and treatment uptake as well as individual and service transformation [6]. This type of model allows for peers to independently and strategically provide client support as equal program partners while negating the organisational barriers that often arise in highly regulated priority care environments [6]. It is imperative to examine the ways in which peer workers (PWs) can give voice to the concerns of people living with HCV, decrease their suspicion of medical professionals, assist in navigating a complex health system, and promote the uptake of DAA treatments. PWs who have life experience with HCV are important health care team members as they can help prospective patients overcome reluctance to enter care [7]. One such example is the LHCF project operating in New South Wales, Australia.

## Case presentation

### Project description

**Fig. 1:**
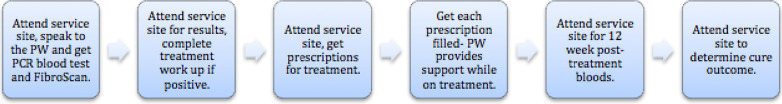
The six-step process to access HCV care through the LHCF program

The core principle guiding the LHCF project is meeting people where they’re at with the health care they need. The LHCF project facilitates access to HCV testing and treatment for individuals who have had difficulty accessing the mainstream system, in priority settings such as residential rehabilitation centres, homelessness services, opioid treatment program clinics and drug health services, mental health services, community health settings, and other outreach settings. The project is a partnership between the community organisation Hepatitis NSW and government-funded clinical services in each health district across the state. The long-term aim of the LHCF project is service-based micro-elimination of HCV achieved by a PW and nurse attending a service site on a regular basis. Hepatitis NSW recruits, trains, pays, and supports PWs to work alongside clinical nurses to engage and support clients over the course of the HCV testing and treatment process. PWs are vital in encouraging clients to be tested, addressing misunderstandings about current HCV treatments, working with the clinical nurses, and listening to and advocating for clients. PWs provide support and facilitate a bridge to health care for people who might already have considerable barriers to accessing the health care they need. All PWs must have a personal history of living with HCV and experience with HCV treatment. Through the full provision of on-site clinical testing options, PWs are able to guide clients to accessing HCV cure in six steps as opposed to nearly double in traditional care pathways (Fig. 1). In this model, the individual does not make an extra appointment or visit; they simply attend the service site (e.g. a methadone clinic) as usual. If reinfection occurs or treatment is unsuccessful, PWs and nurses can continue to support clients and provide retreatment and health care services.

### Project implementation

Since the start of the project in January 2018, LHCF has provided 247 service visits by PWs to key services across the state of NSW, educating and engaging 4,515 people with or at risk of HCV. On-site partner nurses then saw 1,582 of these people and screened 1,045 for HCV and liver health. From 2018 to the end of June 2020, 249 people accessed HCV treatment, while 528 people were referred onward for further health care (e.g. for cirrhosis, hepatitis B, HIV, or other health issues). The LHCF project is adapted to suit the unique context of each service it operates in. This might be a two-week ‘blitz’ type of service, while at other times it is offered weekly or fortnightly for more than 12 months. This all depends on the service, availability of clinical support, and the needs of the clients.

Regular outreach is preferred as this enables PWs to build significant rapport with clients over regularly scheduled visits. PWs offer pre-treatment, on-treatment, and post-treatment support and work with nurses to advocate for and address clients’ needs, provide ongoing support in encouraging access to health care, and work to reduce HCV stigma in the service. This project aligns with recent suggestions that community awareness campaigns should be targeted to raise awareness among socially marginalised groups such as people who inject drugs [5]. The spreading of awareness by word of mouth is especially important in counterbalancing false beliefs from the interferon era, particularly stories of major HCV treatment side effects and suboptimal efficacy [5, 8].

## Discussion and conclusions

### Fundamental attributes of the LHCF project: PW expertise and partnership

Despite how integral people who inject drugs have been to the establishment and ongoing success of harm reduction efforts, their formal and full involvement within such programs is often limited, especially in more medicalised settings. The literature suggests that benefits of peer involvement in HCV prevention and treatment programs include improved client attendance [9] and compliance with medical recommendations [10], improved retention of particular subgroups in HCV treatment (i.e. people who inject drugs) [11], and improved relationships between clients and health care workers [7]. PWs are central to engaging clients who may have had previous negative experiences of health care and are distrustful of health professionals [7].

In LHCF, all PWs are trained, managed, paid, and supported by Hepatitis NSW to utilise their lived experience professionally to engage clients towards receiving HCV testing, treatment, and care. The PWs aim to build rapport with clients through regular interactions and conversation. Importantly, the PWs provide non-judgmental support for clients who might be apprehensive about seeking medical attention due to mistrust, misinformation, and/or stigmatisation within health care settings [12].

The strong PW and clinical nurse partnership is a key part of the LHCF project design. PWs aim to work in a collaborative, professional partnership with the clinical nurses to deliver optimal HCV health care. Through this joint pursuit and a person-centred approach, the PW and clinical nurse plan how to effectively guide and treat prospective clients. The LHCF project encourages clinical nurses to recognise that the PWs are experts on living with HCV and to engage PWs as equal and important care-delivery partners. PWs are available to augment the work of the clinical care team by providing support for people in treatment, communicating reminders from the nurse, and offering advice on how clinical staff can best meet the individual client needs. The LHCF project supports the notion that individuals with non-clinical expertise are able to effectively contribute towards providing optimal HCV care in partnership with clinical staff.

### Addressing misconceptions

The recent development of DAAs has revolutionised the field of HCV treatment and sparked worldwide elimination efforts [13]. Since March 2016, DAAs have been universally available through Australia’s national Medicare system [14]. The extensive research on the efficacy of DAA therapies has led to their promotion as safe and highly tolerable pharmaceuticals [15]. Although the national uptake of DAAs has been noteworthy, misconceptions of DAAs are still a barrier to key populations accessing HCV treatment. A recent study among people who inject drugs by Bryant et al. [8] revealed that 37% of participants had not initiated HCV treatment due to worries about possible side effects. Criticisms of post-interferon era public health interventions suggest that socially marginalised and stigmatised groups can be suspicious of HCV treatment care providers, technologies, and side effects [8].

PWs have a key role in dispelling myths about HCV testing and treatment such as out-dated treatments, treatment access, and reinfection that may contribute to client’s initial reluctance to receiving testing and/or treatment. An important facet of the LHCF project is that the PWs are able to utilise their personal, lived experience to connect with clients to acknowledge and explore the myriad social factors that impact the client’s health motivations and choices and mitigate fear of stigma in health care settings. For example, and following the literature, the LHCF PWs assume that clients may be sceptical or wary of biomedical claims about DAA treatment and can draw on the sharing of lived-experience which is essential to overcoming barriers to diagnosis and linkage to care. PWs can have a key role in dispelling myths about HCV testing and treatment such as out-dated treatments, treatment access, and reinfection that may contribute to a client's initial reluctance to receiving testing and/or treatment.

### Limitations: support for scale-up and sustainability

Funding of the LHCF project is through the organisational base grant provided to Hepatitis NSW by the State Government. The funding arrangement requires the delivery of a range of hepatitis information, resources, and services to support the achievement of state elimination targets. The demand for LHCF project is far greater than resourcing allows. The project is offered free to local health districts to enhance their capacity to engage marginalised people in HCV care and treatment and contribute to local HCV elimination efforts. The health district incurs costs for the time of the clinical nurse who accompanies the PW to outreach sites. The majority of LHCF project’s funding goes to pay the wages of staff and PWs, and while the cost of a PW’s time is dramatically less than a clinical nurse, it adds up with more than 25 PWs, some working weekly or fortnightly, across the state. Each health district is offered an equal amount of peer work annually without cost. The PW team has considerable state-wide capacity, though this may not always meet the demand from each district for peer work. On-site training is provided to the team and in providing effective support to PWs; however, on-site training delivered by project staff is expensive. The LHCF project continues to seek co-funding or an external funding arrangement in order to support health districts in reaching elimination targets.

### The need for additional evaluation

The LHCF project has a substantial amount of evaluation data, qualitative and quantitative, from clinicians involved in the project, which enables open feedback used for specific improvements. The *LHCF Healthcare Worker Survey*, delivered biannually, indicated that having PWs on site has greatly improved clinician-to-client interactions (87.5%) and clinical service delivery (75%) overall. However, the under-funded nature of peer programs as well as the criminalised nature of illicit drug use makes the formal documentation of peer-to-peer quality interactions difficult [16]. While the LHCF project does undertake patient-reported experience measures (PREMs) to survey clients who have engaged with a PW, the survey tool is neither evidence-based nor clinically developed. Therefore, the quantitative and qualitative PREMs evaluation data are limited. Lastly, it may be difficult to measure the true impact of the PW’s engagement efforts as clients may seek HCV health care elsewhere or after the LHCF project has left a service site. The LHCF project aims to address this issue in its next annual evaluation.

## Conclusions

This commentary highlights the value of an interdisciplinary, peer-driven HCV care model in engaging at-risk and marginalised populations in NSW. PWs have an important role in championing innovative testing strategies in HCV care to help facilitate testing uptake [17]. The LHCF project highlights the benefits of having trustworthy, person-centred, and convenient health services to engage and support vulnerable populations throughout HCV testing and treatment, particularly individuals who have been disconnected from the health system. The adaptability of the LHCF project takes into account a client's broader life experiences and their potential points of connection, engagement, and access with the health system. Vital to the effectiveness of the LHCF project is the sharing of lived experience between the PW and client, which is essential to overcoming barriers to HCV treatment. This project has demonstrated that peer-facilitated programmatic approaches can deliver effective rollout of DAA HCV treatments among highly affected and marginalised populations. Establishing ongoing support for scale-up and developing appropriate evaluation methods remain challenges for the LHCF project.

## Data Availability

Not applicable.

## Abbreviations

DAAs: Direct acting antivirals
HCV: Hepatitis C virus
LHCF: Living Hep C Free
NSW: New South Wales
PREMs: Patient-reported experience measures
PW: Peer worker
